# Moving suicide prevention upstream by understanding the effect of flourishing on suicidal ideation in midlife: an instrumental variable approach

**DOI:** 10.1038/s41598-023-28568-2

**Published:** 2023-01-24

**Authors:** Yunyu Xiao, Timothy T. Brown

**Affiliations:** 1 Department of Population Health Sciences, Weill Cornell Medicine, 425 East 61 Street, New York, NY 10065 USA; 2 School of Public Health, University of California, Berkeley, CA USA

**Keywords:** Health care, Medical research, Risk factors, Psychiatric disorders, Trauma, Comorbidities

## Abstract

Prior research has examined the association between flourishing and suicidal ideation, but it is unknown whether this association is causal. Understanding the causality between flourishing and suicidal ideation is important for clinicians and policymakers to determine the value of innovative suicide prevention programs by improving flourishing in at-risk groups. Using a linked nationwide longitudinal sample of 1619 middle-aged adults (mean age 53, 53% female, 88% White) from the National Survey of Midlife Development in the United States (MIDUS), this retrospective cohort study aims to assess the causal relationship between flourishing and suicidal ideation among middle-aged adults in the US. Flourishing is a theory-informed 13-scale index covering three domains: emotional, psychological, and social well-being. Suicidal ideation was self-reported in a follow-up interview conducted after measuring flourishing. We estimated instrumental variable models to examine the potential causal relationship between flourishing and suicidal ideation. High-level flourishing (binary) was reported by 486 (30.0%) individuals, and was associated with an 18.6% reduction in any suicidal ideation (binary) (95% CI, − 29.3– − 8.0). Using alternative measures, a one standard deviation increase in flourishing (*z*-score) was associated with a 0.518 (95% CI, 0.069, 0.968) standard deviation decrease in suicidal ideation (*z*-score). Our results suggest that prevention programs that increase flourishing in midlife should result in meaningful reductions in suicide risk. Strengthening population-level collaboration between policymakers, clinical practitioners, and non-medical partners to promote flourishing can support our collective ability to reduce suicide risks across social, economic, and other structural circumstances.

## Introduction

Suicide is a significant public health crisis in the U.S. From 1999 to 2019, suicide rates among adults aged 45–64 in the U.S. increased by 60%, from 6.0 to 9.6 cases per 100 000 population in females and 43.8% from 20.8 to 29.9 in males^1^. Early intervention and suicide prevention programs are crucial to reduce the risks of suicide^2,3^. Existing suicide prevention efforts are dominated by risk reduction strategies^4,5^. However, meta-analyses have found that common risk factors, including sociodemographic characteristics, psychological factors, physical health, and social relationships, yield limited predictive power for fatal and nonfatal suicidal behavior^6,7^. Besides, suicide risk factors are typically identified through association studies and do not fully account for potential confounding^6^.

With the increasing availability and acceptance of integrative health practices, recent literature has suggested strength-based positive psychological interventions as a promising new approach to preventing suicide^8–11^. These well-being-focused suicide prevention programs can enhance social determinants of health (SDoH) via early intervention among high-risk populations, thus reducing individual and structural burdens of suicidal behaviors.

Flourishing reflects a comprehensive assessment of well-being, with multiple complementary frameworks currently being the subject of research^12–15^. In this study, we focus on Keyes’ framework^15,16^, which combines hedonic and eudaimonic well-being (Table 1). The hedonic framework considers emotional well-being (e.g., happiness, life satisfaction). The eudaimonic framework addresses psychological well-being (e.g., mastery of life, personal growth) and social well-being (e.g., social integration, social cohesion)^13–15^. Flourishing conceptually overlaps with mental well-being and can contribute to a novel approach to reducing suicidal ideation. Recently, human flourishing has been suggested as a conceptual touchstone for prevention-related priorities and objective endpoints in the 2020s^17^. Previous studies have found flourishing associated with a decreased risk of suicidal ideation^18,19^. However, we are unaware of any studies that have assessed the causal effect of flourishing on suicidal ideation.

**Table 1:** Measures of primary exposure (Flourishing Subscales and Domains), primary outcome and instrumental variables.

Constructs	Measures	Coding
Primary exposures: flourishing (measured before suicidal ideation)		
	Items	
Flourishing domain 1: eudaimonic well-being		
*Psychological well-being*		1 Strongly agree; 2 Somewhat agree; 3 A little agree; 4 Neither agree or disagree; 5 A little disagree; 6 Somewhat disagree; 7 Strongly disagree. (R) indicates reverse coding
1. Autonomy	I am not afraid to voice my opinions, even when they are in opposition to the opinions of most people. (R)	
	My decisions are not usually influenced by what everyone else is doing. (R)	
	I tend to be influenced by people with strong opinions	
	I have confidence in my own opinions, even if they are contrary to the general consensus. (R)	
	It’s difficult for me to voice my own opinions on controversial matters	
	I tend to worry about what other people think of me	
	I judge myself by what I think is important, not by the values of what others think is important. (R)	
2. Environmental mastery	In general, I feel I am in charge of the situation in which I live. (R)	
	The demands of everyday life often get me down	
	I do not fit very well with the people and the community around me	
	I am quite good at managing the many responsibilities of my daily life. (R)	
	I often feel overwhelmed by my responsibilities	
	I have difficulty arranging my life in a way that is satisfying to me	
	I have been able to build a living environment and a lifestyle for myself that is much to my liking. (R)	
3. Personal growth	I am not interested in activities that will expand my horizons	
	I think it is important to have new experiences that challenge how you think about yourself and the world. (R)	
	When I think about it, I haven’t really improved much as a person over the years	
	I have the sense that I have developed a lot as a person over time. (R)	
	For me, life has been a continuous process of learning, changing, and growth. (R)	
	I gave up trying to make big improvements or changes in my life a long time ago	
	I do not enjoy being in new situations that require me to change my old familiar ways of doing things	
4. Positive relations with others	Most people see me as loving and affectionate (R)	
	Maintaining close relationships has been difficult and frustrating for me	
	I often feel lonely because I have few close friends with whom to share my	
	I enjoy personal and mutual conversations with family members and friends. (R)	
	People would describe me as a giving person, willing to share my time with others (R)	
	I have not experienced many warm and trusting relationships with others	
	I know that I can trust my friends, and they know they can trust me. (R)	
5. Purpose in life	I live one day at a time and don’t really think about the future	
	I have a sense of direction and purpose in life. (R)	
	I don’t have a good sense of what it is I’m trying to accomplish in life	
	My daily activities often seem trivial and unimportant to me	
	I enjoy making plans for the future and working to make them a reality. (R)	
	Some people wander aimlessly through life, but I am not one of them. (R)	
	I sometimes feel as if I’ve done all there is to do in life	
6. Self-acceptance	When I look at the story of my life, I am pleased with how things have turned out. (R)	
	In general, I feel confident and positive about myself. (R)	
	I feel like many of the people I know have gotten more out of life than I have	
	I like most parts of my personality. (R)	
	In many ways I feels disappointed about my achievements in life	
	My attitude about myself is probably not as positive as most people feel about themselves	
	When I compare myself to friends and acquaintances, it makes me feel good about who I am. (R)	
*Social well-being*		1 Strongly agree; 2 Somewhat agree; 3 A little agree; 4 Neither agree or disagree; 5 A little disagree; 6 Somewhat disagree; 7 Strongly disagree. (R) indicates reverse coding
1. Meaningfulness of society	The world is too complex for me	
	I cannot make sense of what’s going on in the world	
2. Social integration	I don’t feel I belong to anything I’d call a community	
	I feel close to other people in my community (R)	
	My community is my source of comfort (R)	
3. Acceptance of others	People who do a favor expect nothing in return (R)	
	People do not care about other people’s problems	
	I believe that people are kind (R)	
4. Social contribution	I have something valuable to give to the world (R)	
	My daily activities do not create anything worthwhile for my community	
	I have nothing important to contribute to society	
5. Social actualization	The world is becoming a better place for everyone (R)	
	Society has stopped making progress	
	Society isn’t improving for people like me	
Flourishing domain 2: Hedonic well-being		
*Emotional well-being*		
1. Positive affect	During the past 30 days, how much of the time did you feel full of life, happy, cheerful, in good spirits, calm or peaceful, satisfied?	1 All of the time; 2 Most of the time; 3 Some of the time; 4 A little of the time; 5 none of the time
2. Life satisfaction	How would you rate your life overall these days?	0 (the worst possible) to 10 (the best possible)
Primary outcome: suicidal ideation (measured after flourishing)		
Self-reported suicidal ideation from Mood and Anxiety symptoms questionnaire (MASQ)	during the past week, how much you have felt or experienced thoughts about death or suicide?	transformed into binary: have suicidal ideation (a little bit, moderately, quite a bit, or extremely vs. non-suicidal ideation (no)
Instrumental variables: adverse childhood experiences, daily discrimination		
Adverse childhood experiences	Physical abuse, physical neglect, emotional abuse, emotional neglect, sexual abuse, parental divorce, parental depression, and alcohol or drug abuse of any parent	Binary (yes/no)
Daily discrimination	How often on a day-to-day basis do you experience each of the following types of discrimination?	Never, rarely, sometimes, often
	You are threatened or harassed	
	You are called names or insulted	
	People act as if they think you are not as good as they are	
	People act as if they think you are dishonest	
	People act as if they are afraid of you	
	People act as if they think you are not smart	
	You receive poorer service than other people at restaurants or stores	
	You are treated with less respect than other people	
	You are treated with less courtesy than other people	

We used two algorithms to measure flourishing based on the theory. (1) Binary Scoring Algorithm: For each of the 13 scales above (6 psychological + 5 social wellbeing + 2 emotional wellbeing), we computed binary measures, where cut-points were applied to code the upper third of the distribution of each summed scale as one and the lower two-thirds as zero. Flourishing was then indicated when six of the 11 psychological and social well-being scales were equal to one, and at least one of the two emotional well-being scales was equal to one. A second binary measure was constructed in the same way, but omitted the 2 emotional well-being scales such that the measure was equal to one when six of the 11 psychological and social well-being scales were equal to one and zero otherwise. (2) *Z*-score Scoring Algorithm: All 13 scales are summed and converted to a *z*-score. A second measure also summed the measure, but omitted the 2 emotional well-being items, and was then converted to a *z*-score. Flourishing was measured temporally prior to suicidal ideation.

This study is the first to examine the causal effect of flourishing on suicidal ideation. Our design is strengthened by the ability to construct a linked, nationally representative, longitudinal dataset where flourishing was measured temporally before suicidal ideation. To estimate the causal association between flourishing and suicidal ideation^4,20,21^, we conduct instrumental variables (IV) analyses^22–24^. IV is a technique used in health economics, psychiatry, and other areas of medicine to remove parameter bias arising from self-selection, random measurement error, and reverse causation (also called simultaneity bias) when analyzing observational data^25,26^.

**Figure 1:**
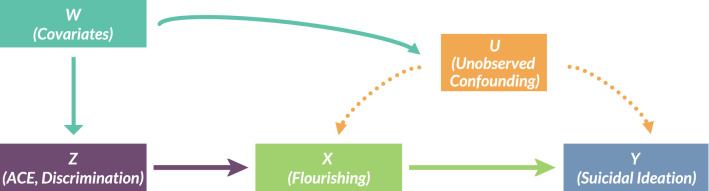
Directed Acyclic Graph.The relationship between flourishing, X, and suicidal ideation, Y, can be estimated using our set of conditional instruments (ACEs, daily discrimination), Z, that are valid conditional on included covariates, W. Y (suicidal ideation) was measured after X (flourishing). W includes severe psychological distress, depression, anxiety, chronic pain, a set of five inflammatory markers, substance use, binge drinking, health status, personality factors, health insurance, and socioeconomic factors (age, sex, race/ethnicity, education, marital status, household income adjusted for household size). The function of Z, conditional on W, in this model is to remove the confounding effects of U (which is a set of unobserved confounders in the error term of the regression analysis) regarding the effect of X on Y. In other words, all these variables in W are included in closing off backdoor paths (non-causal paths) between X and Y, or in econometric terms, to remove any correlation between the set of instruments and the error term.

IV analysis requires the identification of instruments: exogenous variables that influence the factor of interest (primary exposures) but have no direct influence on the primary outcome, conditional on included covariates. We use two valid instruments to determine the causal effect of flourishing on suicidal ideation. These instruments are chosen based on theoretical considerations. The stress-diathesis theory implies that suicidal ideation is due to distal factors (diathesis) from early life and proximal factors from recent stressors^27^. We thus chose two instruments: (1) adverse childhood experiences (ACEs) as a diathesis factor measuring childhood trauma that has been demonstrated to have epigenetic effects^28–31^; and (2) daily discrimination as a stressor factor measuring perceptions of discrimination^32^. Figure 1 shows a directed acyclic graph (DAG) and temporal ordering of primary exposures to outcomes. The key assumption is that both ACEs and daily discrimination are exogenous and should only affect suicidal ideation through their inhibitory impacts on flourishing, conditional on covariates.

### Results

Among 1619 participants, 850 (5.5%) were women, and 769 (47.5%) were men (mean age, 53.4, standard deviation (SD) 12.7 years). Table 2 summarizes the baseline characteristics of our study cohort. 202 participants (12.6%) endorsed suicidal ideation. High-level flourishing (binary) was reported by 486 participants (30%), the mean (SD) of daily discrimination was 12.9 (4.6), and the mean (SD) of ACEs was 3.5 (1.9).

**Table 2:** Characteristics of the participants Included in the study sample (N = 1619)^a^.

Characteristics	N (%)/Mean (SD)
Suicidal Ideation Measures	
Suicidal Ideation (binary), N (%)	202 (12.5%)
Suicidal Ideation, (Range 1–5), Mean (SD)	1.2 (0.5)
Flourishing Measures	
Continuous, (Range:119–406.5), Mean (SD)	311.9 (45.2)
Continuous, (Range:115–392), Mean (SD)	300.7 (43.8)
Binary, N (%)	486 (30.0%)
Binary (eudaimonic only), N (%)	635 (39.2%)
Sociodemographics	
Age, Mean (SD)	53.4 (12.7)
Ages 45–55, N (%)	430 (26.6%)
Ages 55–64, N (%)	393 (24.3%)
Ages 65 and older, N (%)	348 (21.5%)
Female, N (%)	850 (52.5%)
Male, N (%)	769 (47.5%)
Asian/Pacific Islander, N (%)	14 (0.9%)
Black, N (%)	68 (4.2%)
Hispanic, N (%)	62 (3.8%)
Other Race, N (%)	102 (6.3%)
White, N (%)	1431 (88.4%)
Married, N (%)	1124 (69.4%)
Separated, N (%)	26 (1.6%)
Divorced, N (%)	215 (13.3%)
Widowed, N (%)	76 (4.7%)
High School, N (%)	273 (16.9%)
Bachelor's, N (%)	401 (24.8%)
Graduate School, N (%)	435 (26.9%)
Household Income (equivalized) $1s, Mean (SD)	83,874.3 (66,919.6)
Insurance and Health	
Health Insurance, N (%)	1509 (93.2%)
Poor/Fair Health Status, N (%)	170 (10.5%)
Chronic Pain, N (%)	586 (36.2%)
Inflammatory Markers, Mean (SD)	
C-reactive protein, (ug/ml) (range: 0.05–79.3)	2.8 (4.8)
Fibrinogen, (mg/dl) (range: 45–759)	340.7 (79.0)
Interleukin-6, (pg/ml) (range: 0.06–145.05)	1.2 (4.0)
E-Selectin, (ng/ml) (range: 0.09–175)	40.7 (20.2)
Intercellular adhesion molecule-1, (ng/ml) (range: 30–3334)	278.9 (143.8)
Psychological/Mental Health Measures	
Neuroticism (range: 1–4)	2.0 (0.6)
Conscientiousness (range: 1–4)	3.4 (0.5)
Severe Psychological Distress, N (%)	44 (2.7%)
Generalized Anxiety Disorder, N (%)	36 (2.2%)
Depression, N (%)	157 (9.7%)
Substance/Alcohol Abuse	
Substance Use, N (%)	
5 + Drinks (monthly frequency), mean (SD) (range: 0–30)	0.6 (2.8)
Instrumental Variables	
Adverse Childhood Experiences, mean (SD) (range: 0–8)	3.5 (1.9)
Daily Discrimination, mean (SD) (range: 9–33)	12.9 (4.6)

*SD* Standard Deviation.

^a^Data were compiled from the MIDUS 2, MIDUS Refresher, MIDUS Refresher Biomarker Project, and MIDUS 2 Biomarker Project.

Standard overidentification tests were performed using the two-stage limited information maximum likelihood (2SLIML) model. Hansen’s *J* statistic (a test of the joint null hypothesis that the instruments are uncorrelated with the error term and correctly excluded from the estimated equation, assuming at least one instrument is valid) showed that the null hypothesis could not be rejected in any 2SLIML specification.

The strength of the set of instruments could only be tested using the 2SLIML model. The Montiel-Olea-Pflueger weak instrument test^33^, a test that is robust to both heteroskedasticity and serial correlation, yielded an effective *F*-statistic of 15.52, greater than the relevant critical value of 8.38, indicating less than 5% worst-case bias. Hansen’s *J* ${(\chi }^{2}=0.03, p=0.87)$ failed to reject the hypothesis that no instrument was correlated with the error term, given that the other instrument is valid. Finally, an endogeneity test ${(\chi }^{2}=8.69, p=0.003)$ rejected the hypothesis that flourishing is exogenous. See Table S1 in the Supplementary.

The effect of achieving the standard binary definition of flourishing using the bivariate probit model is a 18.6% reduction (95% CI: 8.0, 29.3) in binary suicidal ideation (Table 3, Table S2 in Supplementary). This is not statistically different from the 44.7 percentage point reduction (95% CI: 11.5, 77.8) in binary suicidal ideation when the 2SLIML model is used.

**Table 3:** Effect of Flourishing on Suicidal Ideation^a^.

Measures	Marginal Effect	95% CI		*P* value
Flourishing (Hedonic and Eudaimonic)				
Binary Flourishing, Binary Suicidal Ideation				
Instrumental Variables 2SLIML	− 0.447	− 0.778	− 0.115	0.008
Bivariate Probit	− 0.186	− 0.293	− 0.08	0.001
Continuous Flourishing (*z*-score), Binary Suicidal Ideation				
Instrumental Variables 2SLIML	− 0.228	− 0.4	− 0.06	0.009
Instrumental Variables Probit	− 0.22	− 0.382	− 0.057	0.008
Binary Flourishing, Continuous Suicidal Ideation (*z*-score)				
Instrumental Variables 2SLIML	− 0.959	− 1.84	− 0.079	0.033
Continuous Flourishing (*z*-score), Continuous Suicidal Ideation (*z*-score)				
Instrumental Variables 2SLIML	− 0.518	− 0.968	− 0.069	0.024
Abbreviated Flourishing (Eudaimonic Only)				
Binary Flourishing, Binary Suicidal Ideation				
Instrumental Variables 2SLIML	− 0.414	− 0.737	− 0.091	0.012
Bivariate Probit	− 0.247	− 0.38	− 0.115	< 0.001
Continuous Flourishing (*z*-score), Binary Suicidal Ideation				
Instrumental Variables 2SLIML	− 0.231	− 0.409	− 0.053	0.011
Instrumental Variables Probit	− 0.222	− 0.39	− 0.053	0.01
Binary Flourishing, Continuous Suicidal Ideation (*z*-score)				
Instrumental Variables 2SLIML	− 0.928	− 1.767	− 0.089	0.03
Continuous Flourishing (*z*-score), Continuous Suicidal Ideation (*z*-score)				
Instrumental Variables 2SLIML	− 0.523	− 0.984	− 0.062	0.026

*2SLIML* two-stage limited information maximum likelihood.

^a^Controlled variables include age, sex, race/ethnicity, marital status, education, household income, health insurance, personality factors, substance use, binge drinking, health status, generalized anxiety, depression, severe psychological distress, 5 inflammatory markers, and chronic pain.

Our sensitivity analyses yielded additional findings. We find that the hedonic aspect, while important conceptually, does not make a difference in terms of reducing binary suicidal ideation when using a binary flourishing variable. This may be a special case only relevant to suicidal ideation that may not apply in other applications. The relevant statistics for the 2SLIML model that uses a binary flourishing variable that omits the hedonic scales (these statistics are not available for bivariate probit models) are as follows: Montiel-Olea-Pflueger Effective *F* 15.46 > 7.7 critical value; Hansen’s *J* ${\chi }^{2}=0.36,$ *p* = 0.55; endogeneity ${\chi }^{2}=7.91,$ *p* = 0.005). See Table S1 in the Supplementary. As shown in Table 3, there is no statistical difference in the results when this is done.

In addition, as shown in Table 3, the continuous version of flourishing yields a similarly-size parameter, whether or not the hedonic aspects are included and whether or not 2SLIML or instrumental variables probit is used as an estimator. The relevant test statistics for the 2SLIML are as follows (this set of statistics is not available for the instrumental probit model): continuous flourishing (hedonic and eudaimonic): Montiel-Olea-Pflueger Effective *F* 16.83 > 5.14 critical value; Hansen’s *J* ${\chi }^{2}=0.39,$ *p* = 0.53; endogeneity ${\chi }^{2}=6.68,$ *p* = 0.1; continuous flourishing (eudaimonic only): Montiel-Olea-Pflueger Effective *F* 16.04 > 4.97 critical value; Hansen’s *J* ${\chi }^{2}=0.60,$ *p* = 0.44; endogeneity ${\chi }^{2}=6.61,$ *p* = 0.01.

Further down in Table 3, the binary version of the flourishing combined with the z-score of suicidal ideation yields similarly sized parameters, whether or not the hedonic aspects are included, with suicidal ideation being reduced 0.959 standard deviations (95% CI: 0.079, 1.840) when flourishing is present (Montiel-Olea-Pflueger Effective *F* 15.53 > 8.38 critical value; Hansen’s *J* ${\chi }^{2}=0.45,$ *p* = 0.50; endogeneity ${\chi }^{2}=5.31,$ *p* = 0.02). The corresponding parameters when only the eudaimonic aspects of flourishing are included is 0.928 (95% CI: 0.089, 1.767) (Montiel-Olea-Pflueger Effective *F* 15.55 > 7.67 critical value; Hansen’s *J* ${\chi }^{2}=0.002,$ *p* = 0.96; endogeneity ${\chi }^{2}=5.44,$ *p* = 0.02). See Supplementary Table S5.

Finally, in Table 3, the *z*-score version of flourishing combined with the *z*-score of suicidal ideation yields similarly sized parameters, whether or not the hedonic aspects are included, with the reduction being approximately half a standard deviation when flourishing increases by one standard deviation: − 0.518 (95% CI: − 0.968, − 0.069) (Montiel-Olea-Pflueger Effective *F* 16.83 > 5.09 critical value; Hansen’s *J* ${\chi }^{2}=0.000,$ *P* = 0.99; endogeneity ${\chi }^{2}=4.05,$ *p* = 0.04). The corresponding parameter when only the eudaimonic aspects of flourishing are included is − 0.523 (95% CI: − 0.984, − 0.062) (Montiel-Olea-Pflueger Effective *F* 16.04 > 4.92 critical value; Hansen’s *J* ${\chi }^{2}=0.01,$ *p* = 0.92; endogeneity ${\chi }^{2}=4.07,$ *p* = 0.04). See Table S6 in the Supplementary. All of the above models correct for measurement error and omitted variable bias^34–36^.

## Discussion

This is the first study to clarify the nature of the association between flourishing and suicidal ideation. Using the IV approach, we corrected for bias in the estimated parameters of flourishing due to omitted variables or measurement error^35^. Reverse causation was ruled out by the temporal ordering of the data (suicidal ideation was measured after flourishing). Our findings demonstrated the negative associations of ACEs and discrimination with flourishing^37–39^, as well as the effect of flourishing on reducing suicidal ideation^18,19^. Results are larger in magnitude but consistent with previous studies, most of which used cross-sectional designs. Flourishing inhibits suicidal ideation both when defined as a threshold to achieve and as a continuous set of measures to improve. Flourishing also need not include hedonic measures, at least in this application. 

The first major strength of our investigation is the use of instrumental variables analysis to account for unmeasured confounding variables^40,41^. We determined that ACEs and daily discrimination are negatively correlated with flourishing at approximately the same magnitudes (when z-scores are used), suggesting that stress and diathesis factors damage flourishing similarly. Emerging literature, based on the cross-sectional National Survey of Children’s Health, has revealed the negative impact of ACEs on childhood flourishing^42,43^. Similarly, others have shown an association between daily discrimination and flourishing^17^. We extended such dose–response relationships in the context of the U.S. midlife population. Existing positive psychology and human flourishing theories can explain our findings. People who are flourishing will thrive amid adversity by maximizing their potential by changing abilities and limitations^14^, which reduces the risks of suicidal ideation. By contrast, those who lack purpose in life (psychological aspect of flourishing) are more likely to consider suicide when exposed to childhood trauma or daily discrimination^14^.

The causal effect of flourishing on reducing suicidal ideation suggests flourishing can serve as a target of suicide prevention, shifting the paradigm from the traditional notion of risk reduction towards a more holistic approach—focusing on wellbeing and social determinants of health (SDoH) including flourishing, especially by enhancing social networks and social connectedness^17^. Our second major contribution is to parse the key constructs of flourishing to those that are modifiable and can reduce suicidal ideation via clinical or population-level interventions. Using multiple flourishing measures, we found that the hedonic aspect of flourishing does not add additional magnitude to the measured relationship compared with the situation when we only included the eudaimonic aspects of flourishing. While this may not be the case for other outcomes, this is nevertheless an important issue, as the eudaimonic aspects of flourishing are more modifiable and can be taught through direct interventions (aimed at improving psychological and social well-being measured in our study). Improving the eudaimonic aspects of flourishing may improve the hedonic aspects of flourishing^44^. Evidence-based positive psychology interventions (focusing on systematically promoting mindfulness, patient-caregiver dyadic interpersonal interactions, and coping) have been associated with increased positive affect and reduced depression and mortality in medical populations^10,12^.

Flourishing-based suicide prevention can be useful and effectively implemented in both clinical care and population-based settings^12,12^. Within psychiatry, screening for flourishing may be a promising way to detect individuals susceptible to childhood trauma or stress that could end in suicide. Treating patients using the lens of flourishing may help clinicians provide better patient-centered care that is more holistic and more acceptable to patients^42,45^. Flourishing-centered suicide prevention programs may be especially relevant to suicide attempts encountered in the emergency department setting given the high suicide rates of 178 per 100 000 person-years in the first three months after discharge and the lack of care coordination with the warm handoff^46,47^. Family-focused interventions, as promoted by the Institute of Medicine, may be pivotal to promoting flourishing by supporting family connections^37,48^. Equally important is to promote flourishing among physicians and healthcare workers who may encounter patients with suicidal ideation, as improving the personal growth and environmental mastery of physicians and healthcare workers may reduce their risks of burnout^49^.

At the *population* level, flourishing-based suicide prevention could be achieved effectively with evidence-based programs and policy efforts across the sectors of health care, education, and human services^17^. On the one hand, it is key to implement a collaborative and holistic approach to address structural factors (e.g., policies that improve education, income, reduce racism and other forms of discrimination, etc.), create safe and connected families, and build connected communities as a foundation for intergenerational flourishing. On the other hand, our findings highlight that the broad dissemination of individual-oriented “positive psychology” interventions (e.g., strength-based asset development, emotional regulations, positive interpersonal skills)^12,13^ can reduce suicide via a more effective public health approach. These individually-oriented interventions can amplify the positive effects of other policies focused on structural change. Our findings emphasize the importance of flourishing in reducing suicidal ideation as an urgent consideration with potential short-term and longer-term benefits.

### Limitations

Our study had limitations. First, studies of attrition and retention in MIDUS have revealed that White, female, married people with higher education and better health were more likely to stay in subsequent waves^50^. Given the possible disproportionate exposure to ACEs and discrimination among vulnerable populations (e.g., racial/ethnic minorities, males), this attrition bias suggests that our findings may be understated. However, the recently NIA-funded MIDAS Retention Early Warning project reinstated a substantial portion of dropouts and provided valuable opportunities to investigate the extent to which flourishing among those who dropped out would cause reduced suicidal ideation^43^. Second, the measure of suicidal ideation was self-reported, which may underestimate suicide risks. In addition, we only used a single question to measure suicidal ideation. Suicidal ideation is a multifaceted issue, and there are full instruments developed for this single issue, but to our knowledge, such instruments are not available in data that also contains measures of flourishing. Third, our operationalization of flourishing is relatively narrow, whereas alternative measures of flourishing cover a broader array of life domains. Future studies are encouraged to validate the findings in this study using a more diverse sample and other measures of flourishing. Lastly, although we included all common suicide risks in our data, results may be biased if some risk factors for suicide that correlate with our instruments are unmeasured.

In sum, risk-reduction suicide interventions have focused on teaching people how not to die, whereas flourishing-focused suicide prevention teaches us how to *live*. Our evidence showing the possible causal effect of promoting flourishing on reducing suicidal ideation provides a promising future direction for a more effective and scalable approach.

## Methods

### Data and participants

We obtained data from four samples of the National Survey of Midlife Development in the United States (MIDUS) study that allowed us to construct two longitudinal cohorts, which we then combined^51^. The first is the MIDUS 2 Project (2004–2006)^52^, which contains a 10-year follow-up of participants from the original MIDUS study. The second is the MIDUS 2 Biomarker Project (2004–2009)^53^, a longitudinal follow-up of MIDUS 2, containing biological assessments and information on suicidal ideation. The third is the MIDUS Refresher Study (2011–2014)^54^, designed to replenish the MIDUS cohort. The fourth is from the MIDUS Refresher Biomarker study (2012–2016)^55^, which paralleled the MIDUS 2 Biomarker Project and was a longitudinal follow-up of the MIDUS Refresher Study, containing biological assessments and information on suicidal ideation. All participants gave informed consent^56^. The response rates for the four samples were 81.0%, 39.3%, 73.0%, and 41.5%, respectively. Following previous research^40,57^, the current study linked these four samples (*n* = 1619). We followed the Strengthening the Reporting of Observational Studies in Epidemiology (STROBE) reporting guidelines. This study was not considered human subjects research by the Committee for the Protection of Human Subjects at the University of California, as defined by federal regulations at 45 CFR 46.102 (DHHS) and/or 21 CFR 50.3 (FDA).

### Measures

The primary outcome, primary exposure, and instrumental variable described below have a clear temporal ordering (Fig. 1). Suicidal ideation is temporally preceded by flourishing, which is temporally preceded by the set of instrumental variables (all of which look back to exogenous events from the past).

#### Primary outcome: suicidal ideation

Suicidal ideation is a single question from the Mood and Anxiety Symptoms Questionnaire (MASQ) available in both the MIDUS 2 Biomarker and Refresher Biomarker projects^58^, asking the respondent, “during the past week, how much you have felt or experienced thoughts about death or suicide?” Because we want to capture any amount of suicidal ideation, we transformed this question into a binary variable (having “a little bit” up to “extreme” thoughts of suicide) vs. non-suicidal ideation cases (having “no” thoughts of suicide). Binary measures of suicidal ideation are commonly employed when measuring self-report suicidal ideation using similar measures of MASQ like PHQ-9^59^ (e.g., dichotomizing the 4-point scale responses^60,61^) and have been found to be valid for clinical assessment^41^. For sensitivity analyses, following a recent review regarding the effectiveness of different suicide screening measures^62^, we transformed the original suicidal ideation question into a *z*-score, which is expressed in standard deviations and avoids any loss of data.

#### Primary exposure: flourishing

Flourishing is based on a 13-scale index measuring emotional well-being (hedonic aspect), psychological well-being, and social well-being (eudaimonic aspects) (Table 1)^15^.

*Emotional well-being* contains two subscales. These include a 6-item measure of positive affect, with each item being measured on a 5-point scale (1 = none of the time, 2 = a little of the time, 3 = some of the time, 4 = most of the time, 5 = all of the time), and a single life satisfaction item measured on a 10-point scale (1 = worst possible life overall these days to 10 = best possible life overall these days).

*Psychological well-being* includes 6 subscales. Each item is coded on a 7-point scale (1 = strongly disagree to 7 = strongly agree). Items are reverse coded as indicated in Table 1.

*Social well-being* includes 5 subscales, one with 2 items and the remaining each having 3 items. Each item is scored on a 7-point scale and coded in the same way as the items for psychological well-being. Items are reverse coded as indicated in Table 1.

Keyes’ binary measure of flourishing (high-level flourishing) is constructed using the following algorithm: for each of the 13 scales described above, we computed binary measures, where cut-points were applied to code the upper third of the distribution of each scale as one, and the lower two-thirds as zero. Flourishing was then indicated when six of the 11 psychological and social well-being scales were equal to one, and at least one of the two emotional well-being scales was equal to one^16^.

**Figure 2:**
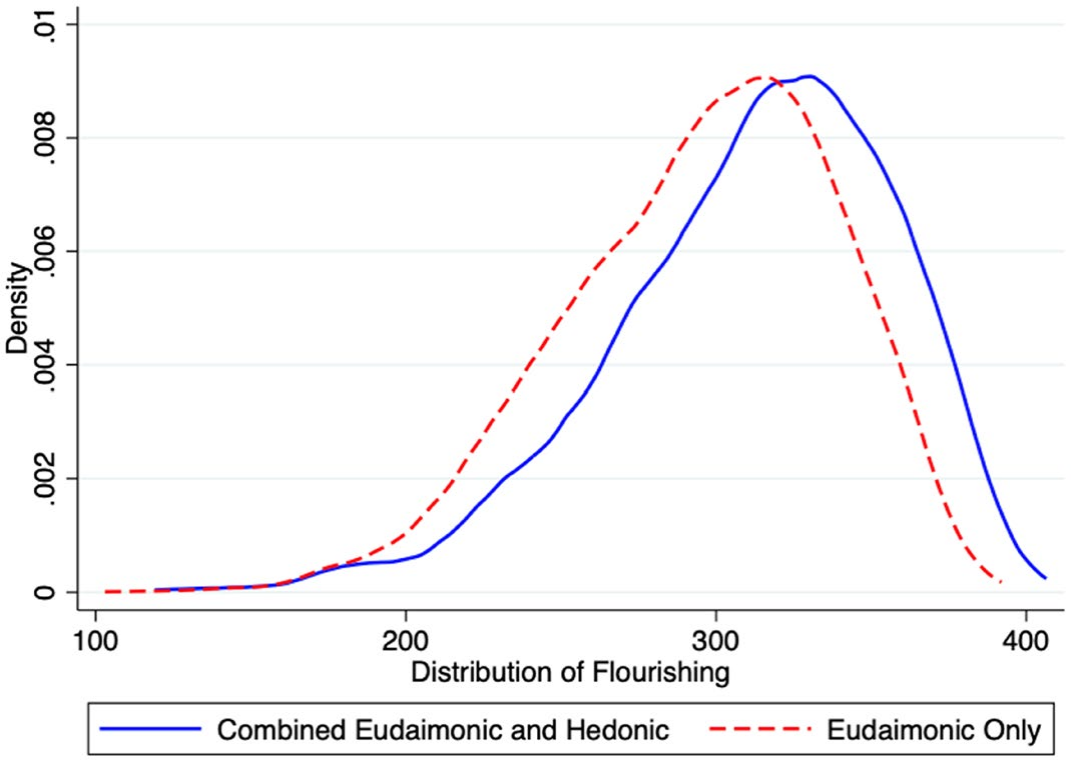
Distribution of Flourishing (Continuous Measures).

To facilitate sensitivity analyses, we also constructed additional flourishing measures. These alternative measures are designed to determine whether outcomes are sensitive to the inclusion/exclusion of the hedonic aspect of flourishing and whether flourishing may be not only be considered as a state (whether an individual is in the upper third of emotional, psychological, and social well-being), but also as a process (improvements in emotional, psychological, and social well-being are valuable and desirable as one moves toward the upper one-third threshold goal). We thus constructed an alternative binary measure that omitted the two emotional well-being (hedonic) scales. We also constructed two continuous measures of flourishing (one that included both the hedonic and eudaimonic scale sets, and one that only included the eudaimonic scale set) by summing all relevant scales and converting the sum to a *z*-score^15–17^. The distribution of each continuous measure is approximately normal. See Fig. 2. These scales are described in Table 1, and the relevant scoring algorithms are described in the footnotes to the table.

#### Instrumental variables: ACEs and daily discrimination

Exposure to ACEs is a summed score of 8 binary (yes/no) categories of adverse experiences (physical abuse, physical neglect, emotional abuse, emotional neglect, sexual abuse, parental divorce, parental depression, and alcohol or drug abuse of any parent) occurring before age 18 (range 0–8, Table 1). This measure has been validated and is consistent with empirical applications^29,37,63^ and theory^28^. We transformed this scale into a *z*-score.

Daily discrimination is measured using the validated daily perceived discrimination scale (Table 1)^32,64^. Responses to each question are coded 0–3 (never, rarely, sometimes, often) and then summed. We transformed this scale into a *z*-score.

#### Covariates

We controlled for potential pathways between the instrumental variables and suicidal ideation to ensure that the instruments only affect suicidal ideation through their impact on flourishing. We thus include the following known risk factors for suicidal ideation: (1) socioeconomic characteristics, including participants’ age, sex, race/ethnicity, marital status, educational level, household income (adjusted for household size), and health insurance status; (2) physical health, including diminished health status (poor health or fair health), binge drinking (number of days with more than five drinks per day in the past 30 days), substance use (ever used the following in the past 12 months either without a doctor's prescription, in larger amounts than prescribed, or for a longer period than prescribed: sedatives, tranquilizers, stimulants, painkillers, antidepressants, inhalants, marijuana/hashish, cocaine/crack, LSD/hallucinogens, heroin), any chronic pain (yes/no), and a set of 5 inflammatory markers, which have been found to be affected by ACEs and could impact suicidal ideation^40,65^, including C-reactive protein, interleukin-6, fibrinogen, E-selectin, and intercellular adhesion molecule; (3) psychological factors, including the Kessler K6^66^, generalized anxiety disorder^67^, depressed affect^67^; and 4) two personality traits measured by the Big Five (neuroticism, conscientiousness) that are known determinants of suicidal ideation^68–70^.

### Statistical analysis

#### Main analysis

Our main analysis includes three instrumental variables models: a two-stage limited information maximum likelihood (2SLIML) model, a bivariate probit model that is a recursive probit model^71–73^ and an IV probit model that is a control function^35^. All instrumental variables models must satisfy three criteria: instruments must be exogenous, strongly correlate with the endogenous variable of interest, and not correlate with the error term^24^.

Both instruments were theoretically exogenous: ACEs occurred in childhood, and daily discrimination occurred due to the actions of third parties. We evaluated whether or not the correlation between the endogenous variable of interests (flourishing) and instruments (ACEs and daily discrimination) is sufficiently strong using the Montiel-Olea-Pfluegar weak instrument test^33^. To avoid any potential correlations between the instruments and the error term, we included the known risk factors for suicidal ideation, including socioeconomic characteristics (e.g., marital status, household income), physical health (e.g., chronic pain, inflammation), psychological factors, and social relationships, which are listed in detail above^6,47,74,75^. We additionally evaluated whether the instruments were exogenous using an overidentification test and whether the flourishing parameter was significantly different after being corrected (endogeneity test). Figure 1 presents a directed acyclic graph (DAG) of the model^76,77^. All models are estimated using Stata 16^33,78^.

#### Sensitivity analysis

We performed three sets of sensitivity analyses. We used binary measures of flourishing that omitted emotional well-being (measures included eudaimonic aspects only). We used continuous versions of flourishing (*z*-scores) for both the eudaimonic-hedonic measure of flourishing and the eudaimonic-only measure of flourishing. Finally, we estimated every model substituting the *z*-score of suicidal ideation for the binary version. Thus, we estimated all combinations of measures of suicidal outcomes (binary, *z*-score) and measures of flourishing (binary eudaimonic-hedonic, binary eudaimonic only, continuous eudaimonic-hedonic, continuous eudaimonic only). Binary suicidal ideation-binary flourishing models were estimated using 2SLIML and bivariate probit; binary suicidal ideation-continuous flourishing models were estimated using 2SLIML and IV probit (control function probit); continuous suicidal ideation-binary flourishing models were estimated using 2SLIML, and continuous suicidal ideation-continuous flourishing models were estimated using 2SLIML.

### Ethical approval

This study was not required to obtain ethics approval since it uses publicly available data that contains no identifiable private information and is therefore not considered human subjects research by the Committee for the Protection of Human Subjects at the University of California. The authors did not have access to any personally identifiable information or information that would link the data to individuals’ identities. All data are reported in aggregate to eliminate the possibility of deductive identification of individuals.

## Supplementary Information

Supplementary Information.

## Data Availability

Data sharing: Data are accessible through Inter-university Consortium for Political and Social Research (ICPSR). The public-use data files in this collection are available for access by the general public through https://www.icpsr.umich.edu/web/ICPSR/series/203. Access does not require affiliation with an ICPSR member institution. All Stata code is accessible from the corresponding author.
