# Supplementary material for: Moving suicide prevention upstream by understanding the effect of flourishing on suicidal ideation in midlife: an instrumental variable approach
Source: Sci Rep. 2023 Jan 24;13:1320. doi: 10.1038/s41598-023-28568-2 (PMC9873734; doi:10.1038/s41598-023-28568-2)
Supplement: Supplementary file 1 — Supplementary Information. [file 41598_2023_28568_MOESM1_ESM.docx]

Supplementary Materials

[Supplementary Table S1 Effect of Flourishing (binary) on Suicidal Ideation (binary) - Linear Models 2](#_Toc121392130)

[Supplementary Table S2 Effect of Flourishing (binary) on Suicidal Ideation (binary) - Nonlinear Model 5](#_Toc121392131)

[Supplementary Table S3 Effect of Flourishing (z-score) on Suicidal Ideation (binary) - Linear Models 8](#_Toc121392132)

[Supplementary Table S4 Effect of Flourishing (z-score) on Suicidal Ideation (binary) - Nonlinear Models 12](#_Toc121392133)

[Supplementary Table S5 Effect of Flourishing (binary) on Suicidal Ideation (z-score) - Linear Models 15](#_Toc121392134)

[Supplementary Table S6 Effect of Flourishing (z-score) on Suicidal Ideation (z-score) - Linear Models 19](#_Toc121392135)

# Supplementary Table S1 Effect of Flourishing (binary) on Suicidal Ideation (binary) - Linear Models

|  | (1) | (2) | (3) | (4) |
| --- | --- | --- | --- | --- |
|  | Flourishing (binary) | Suicidal Ideation  (binary) | Flourishing  (binary) | Suicidal Ideation  (binary) |
| VARIABLES | First Stage | Second Stage | First Stage | Second Stage |
| Binary Flourishing (Eudaimonic and Hedonic) | - | -0.447** | - | - |
|  |  | (0.169) |  |  |
| Binary Flourishing (Eudaimonic only) | - | - | - | -0.414* |
|  |  |  |  | (0.165) |
| Ages 45-54 | -0.002 | 0.053* | -0.005 | 0.053* |
|  | (0.028) | (0.027) | (0.030) | (0.027) |
| Ages 55_64 | 0.046 | 0.030 | 0.038 | 0.026 |
|  | (0.031) | (0.027) | (0.033) | (0.026) |
| Ages 65 and over | 0.071* | 0.072* | 0.023 | 0.051 |
|  | (0.033) | (0.031) | (0.035) | (0.028) |
| Female | 0.041 | 0.011 | 0.048* | 0.012 |
|  | (0.023) | (0.020) | (0.024) | (0.021) |
| Asian/Pacific Islander | -0.147 | -0.120 | -0.170 | -0.128 |
|  | (0.115) | (0.080) | (0.128) | (0.084) |
| Black | 0.050 | 0.050 | 0.029 | 0.033 |
|  | (0.051) | (0.051) | (0.055) | (0.050) |
| Hispanic | 0.110 | 0.064 | 0.097 | 0.055 |
|  | (0.060) | (0.063) | (0.064) | (0.061) |
| Other Race | -0.062 | 0.006 | -0.060 | 0.007 |
|  | (0.042) | (0.051) | (0.045) | (0.049) |
| Married | 0.034 | -0.074* | 0.003 | -0.086* |
|  | (0.034) | (0.036) | (0.036) | (0.035) |
| Seperated | -0.031 | -0.103 | 0.021 | -0.076 |
|  | (0.066) | (0.080) | (0.079) | (0.090) |
| Divorced | -0.028 | -0.100* | -0.061 | -0.111** |
|  | (0.040) | (0.042) | (0.043) | (0.042) |
| Widowed | -0.024 | -0.070 | -0.011 | -0.063 |
|  | (0.059) | (0.057) | (0.060) | (0.057) |
| High School | -0.010 | 0.069 | 0.030 | 0.085 |
|  | (0.056) | (0.057) | (0.063) | (0.057) |
| Some College | 0.050 | 0.073 | 0.104 | 0.093 |
|  | (0.054) | (0.056) | (0.061) | (0.057) |
| Bachelor's Degree | 0.067 | 0.123* | 0.097 | 0.133* |
|  | (0.056) | (0.059) | (0.063) | (0.060) |
| Graduate School | 0.066 | 0.087 | 0.168** | 0.126 |
|  | (0.057) | (0.059) | (0.064) | (0.065) |
| Equivalized Household Income, $1000s | 0.000* | 0.000 | 0.000** | 0.000 |
|  | (0.000) | (0.000) | (0.000) | (0.000) |
| Health Insurance | -0.019 | 0.016 | -0.073 | -0.007 |
|  | (0.037) | (0.041) | (0.042) | (0.043) |
| Poor or Fair Health Status (binary) | -0.064* | 0.051 | -0.062 | 0.053 |
|  | (0.028) | (0.039) | (0.033) | (0.038) |
| Chronic Pain (binary) | -0.026 | 0.016 | -0.009 | 0.023 |
|  | (0.022) | (0.021) | (0.024) | (0.020) |
| C-reactive protein (z-score) | 0.012 | 0.004 | 0.004 | 0.000 |
|  | (0.011) | (0.010) | (0.010) | (0.009) |
| Fibrinogen (z-score) | -0.005 | 0.007 | -0.009 | 0.005 |
|  | (0.012) | (0.011) | (0.013) | (0.011) |
| Interleukin-6 (z-score) | -0.008 | 0.003 | -0.013** | 0.001 |
|  | (0.005) | (0.007) | (0.005) | (0.007) |
| E-Selectin (z-score) | -0.011 | -0.011 | -0.013 | -0.012 |
|  | (0.012) | (0.010) | (0.012) | (0.010) |
| Intercellular adhesion molecule-1 (z-score) | -0.002 | -0.002 | 0.007 | 0.002 |
|  | (0.011) | (0.010) | (0.010) | (0.010) |
| Neuroticism | -0.199*** | -0.066 | -0.216*** | -0.066 |
|  | (0.017) | (0.042) | (0.019) | (0.044) |
| Conscientiousness | 0.154*** | 0.052 | 0.178*** | 0.057 |
|  | (0.021) | (0.035) | (0.023) | (0.038) |
| Severe Psychological Distress (z-score) | 0.095* | 0.215** | 0.058 | 0.193* |
|  | (0.042) | (0.079) | (0.046) | (0.076) |
| Generalized Anxiety Disorder (binary) | 0.093** | -0.006 | 0.065 | -0.022 |
|  | (0.034) | (0.082) | (0.048) | (0.081) |
| Depressed Affect (M2) | 0.011 | 0.130** | -0.000 | 0.125** |
|  | (0.031) | (0.041) | (0.037) | (0.041) |
| 5+ Drinks (binary) | 0.003 | -0.001 | 0.001 | -0.002 |
|  | (0.004) | (0.004) | (0.004) | (0.004) |
| Substance Use (binary) | -0.048 | 0.018 | 0.012 | 0.043 |
|  | (0.028) | (0.032) | (0.032) | (0.030) |
| M2 | 0.011 | 0.020 | 0.037 | 0.029 |
|  | (0.022) | (0.020) | (0.023) | (0.021) |
| ACEs (z-score) | -0.044*** | - | -0.038*** | - |
|  | (0.010) |  | (0.010) |  |
| Daily Discrimination (z-score) | -0.022* | - | -0.040*** | - |
|  | (0.011) |  | (0.012) |  |
| Constant | 0.132 | 0.075 | 0.161 | 0.089 |
|  | (0.105) | (0.110) | (0.115) | (0.109) |
|  |  |  |  |  |
| Montiel-Olea-Pfluegar Weak Instrument Test |  |  |  |  |
| Effective F | 15.52 |  | 15.46 |  |
| Critical Value: 5% Worst Case Bias | 8.38 |  | 7.73 |  |
| Overidentification Test |  |  |  |  |
| Hansen's J (p-value) | 0.025(0.87) |  | 0.359(0.55) |  |
| Endogeneity Test |  |  |  |  |
| Chi-squared (p-value) | 8.69(0.003) |  | 7.91(0.005) |  |
| Observations | 1,619 | 1,619 | 1,619 | 1,619 |
| Robust standard errors in parentheses |  |  |  |  |
| *** p<0.001, ** p<0.01, * p<0.05 |  |  |  |  |
| Two-stage Limited Information Maximum Likelihood (2SLIML) | |  |  |  |

# Supplementary Table S2 Effect of Flourishing (binary) on Suicidal Ideation (binary) - Nonlinear Model

|  | (1) |  | (2) |  | (3) |  | (4) |  |
| --- | --- | --- | --- | --- | --- | --- | --- | --- |
|  | Flourishing  (binary) |  | Suicidal Ideation (binary) |  | Flourishing (binary) |  | Suicidal Ideation (binary) |  |
| VARIABLES | First Stage |  | Second Stage |  | First Stage |  | Second Stage |  |
|  | Bivariate Probit  Coefficients | Standard Errors | Bivariate Probit  Coefficients | Standard  Errors | Bivariate Probit  Coefficients | Standard  Errors | Bivariate Probit  Coefficients | Standard  Errors |
| Binary Flourishing (Eudaimonic and Hedonic) | - | - | -1.080*** | 0.290 | - | - | - | - |
| Binary Flourishing (Eudaimonic only) | - | - | - | - | - | - | -1.242*** | 0.290 |
| Ages 45-54 | 0.002 | 0.113 | 0.260* | 0.126 | 0.014 | 0.107 | 0.236* | 0.121 |
| Ages 55_64 | 0.162 | 0.114 | 0.086 | 0.131 | 0.134 | 0.111 | 0.077 | 0.128 |
| Ages 65 and over | 0.226 | 0.124 | 0.255 | 0.139 | 0.085 | 0.119 | 0.200 | 0.132 |
| Female | 0.150* | 0.076 | 0.020 | 0.091 | 0.158* | 0.078 | 0.037 | 0.090 |
| Asian/Pacific Islander | -0.557 | 1.385 | -0.323 | 2.345 | -0.586 | 0.951 | -0.350 | 2.346 |
| Black | 0.234 | 0.213 | 0.164 | 0.199 | 0.149 | 0.205 | 0.120 | 0.196 |
| Hispanic | 0.449* | 0.226 | 0.205 | 0.244 | 0.330 | 0.213 | 0.202 | 0.235 |
| Other Race | -0.276 | 0.195 | 0.075 | 0.201 | -0.217 | 0.165 | 0.058 | 0.190 |
| Married | 0.121 | 0.142 | -0.317* | 0.135 | 0.013 | 0.119 | -0.321** | 0.128 |
| Seperated | -0.257 | 0.756 | -0.377 | 0.448 | 0.005 | 0.295 | -0.277 | 0.325 |
| Divorced | -0.086 | 0.174 | -0.392 | 0.157 | -0.186 | 0.155 | -0.406 | 0.152 |
| Widowed | -0.062 | 0.230 | -0.216 | 0.231 | 0.007 | 0.210 | -0.193 | 0.224 |
| High School | 0.142 | 0.309 | 0.263 | 0.288 | 0.201 | 0.279 | 0.317 | 0.277 |
| Some College | 0.369 | 0.298 | 0.222 | 0.280 | 0.440 | 0.265 | 0.308 | 0.267 |
| Bachelor's Degree | 0.420 | 0.304 | 0.425 | 0.286 | 0.417 | 0.275 | 0.478 | 0.269 |
| Graduate School | 0.427 | 0.305 | 0.242 | 0.295 | 0.646 | 0.274 | 0.394 | 0.283 |
| Equivalized Household Income, $1000s | 0.000 | 0.000 | 0.000 | 0.000 | 0.000* | 0.000 | 0.000 | 0.000 |
| Health Insurance | 0.058 | 0.185 | 0.083 | 0.174 | -0.181 | 0.150 | 0.003 | 0.174 |
| Poor or Fair Health Status (binary) | -0.321* | 0.160 | 0.202 | 0.132 | -0.212 | 0.143 | 0.182 | 0.127 |
| Chronic Pain (binary) | -0.083 | 0.086 | 0.122 | 0.093 | -0.019 | 0.078 | 0.129 | 0.088 |
| C-reactive protein (z-score) | 0.045 | 0.044 | 0.002 | 0.050 | 0.009 | 0.041 | -0.009 | 0.049 |
| Fibrinogen (z-score) | -0.012 | 0.047 | 0.035 | 0.049 | -0.031 | 0.043 | 0.027 | 0.048 |
| Interleukin-6 (z-score) | -0.080 | 0.107 | 0.009 | 0.159 | -0.093 | 0.094 | 0.002 | 0.155 |
| E-Selectin (z-score) | -0.032 | 0.046 | -0.036 | 0.046 | -0.038 | 0.043 | -0.042 | 0.044 |
| Intercellular adhesion molecule-1 (z-score) | -0.021 | 0.062 | -0.007 | 0.048 | 0.019 | 0.043 | 0.002 | 0.051 |
| Neuroticism | -0.726*** | 0.077 | -0.095 | 0.121 | -0.689*** | 0.070 | -0.153 | 0.117 |
| Conscientiousness | 0.584*** | 0.090 | 0.077 | 0.117 | 0.593*** | 0.082 | 0.137 | 0.114 |
| Severe Psychological Distress (z-score) | -0.294 | 2.719 | 0.522* | 0.250 | -0.423 | 2.072 | 0.444 | 0.244 |
| Generalized Anxiety Disorder (binary) | -4.284*** | 0.395 | -0.109 | 0.291 | -0.241 | 2.346 | -0.120 | 0.277 |
| Depressed Affect (binary) | -0.026 | 0.176 | 0.418 | 0.139 | -0.032 | 0.142 | 0.380** | 0.135 |
| 5+ Drinks (binary) | 0.009 | 0.015 | -0.006 | 0.024 | -0.002 | 0.015 | -0.007 | 0.023 |
| Substance Use (binary) | -0.247 | 0.145 | 0.112 | 0.118 | 0.064 | 0.120 | 0.170 | 0.110 |
| M2 | 0.058 | 0.078 | 0.050 | 0.085 | 0.110 | 0.075 | 0.087 | 0.084 |
| ACEs (z-score) | -0.144*** | 0.034 | - | - | -0.125*** | 0.030 | - | - |
| Daily Discrimination (z-score) | -0.121*** | 0.046 | - | - | -0.160*** | 0.042 | - | - |
| Constant | -1.648*** | 0.463 | -1.327** | 0.500 | -1.328** | 0.424 | -1.237* | 0.497 |
|  |  |  |  |  |  |  |  |  |
| ATE: Binary Flourishing (Eudaimonic and Hedonic) | |  | -0.186*** | 0.054 |  |  | -0.248*** | 0.067 |
| ATE: Flourishing (Eudaimonic only) |  |  |  |  |  |  |  |  |
| Observations | 1,619 |  | 1,619 |  | 1,619 |  | 1,619 |  |
| *** p<0.001, ** p<0.01, * p<0.05 |  |  |  |  |  |  |  |  |
| Bivariate Probit Models |  |  |  |  |  |  |  |  |
| Bootstrapped Standard Errors (500 repetitions) | |  |  |  |  |  |  |  |
| ATE: Average Treatment Effect |  |  |  |  |  |  |  |  |

# Supplementary Table S3 Effect of Flourishing (z-score) on Suicidal Ideation (binary) - Linear Models

|  | (1) | (2) | (3) | (4) |
| --- | --- | --- | --- | --- |
|  | Flourishing  (z-score) | Suicidal Ideation  (binary) | Flourishing  (z-score) | Suicidal Ideation  (binary) |
| VARIABLES | First Stage | Second Stage | First Stage | Second Stage |
| Flourishing (Eudaimonic and Hedonic) (z-score) | - | -0.228** | - | - |
|  |  | (0.088) |  |  |
| Flourishing (Eudaimonic only) (z-score) | - | - | - | -0.231* |
|  |  |  |  | (0.091) |
| Ages 45-54 | 0.014 | 0.058* | 0.015 | 0.059* |
|  | (0.052) | (0.026) | (0.052) | (0.026) |
| Ages 55_64 | 0.054 | 0.021 | 0.050 | 0.022 |
|  | (0.057) | (0.025) | (0.057) | (0.025) |
| Ages 65 and over | 0.156** | 0.077* | 0.147* | 0.076* |
|  | (0.059) | (0.031) | (0.059) | (0.031) |
| Female | 0.132** | 0.023 | 0.129** | 0.022 |
|  | (0.040) | (0.022) | (0.040) | (0.022) |
| Asian/Pacific Islander | -0.075 | -0.075 | -0.069 | -0.075 |
|  | (0.223) | (0.075) | (0.224) | (0.075) |
| Black | 0.100 | 0.052 | 0.101 | 0.043 |
|  | (0.096) | (0.050) | (0.096) | (0.049) |
| Hispanic | 0.116 | 0.040 | 0.107 | 0.040 |
|  | (0.113) | (0.056) | (0.114) | (0.056) |
| Other Race | -0.051 | 0.020 | -0.055 | 0.019 |
|  | (0.080) | (0.048) | (0.081) | (0.049) |
| Married | 0.077 | -0.071* | 0.067 | -0.072* |
|  | (0.065) | (0.036) | (0.065) | (0.036) |
| Seperated | 0.156 | -0.045 | 0.165 | -0.046 |
|  | (0.133) | (0.087) | (0.129) | (0.085) |
| Divorced | -0.023 | -0.091* | -0.025 | -0.090* |
|  | (0.081) | (0.040) | (0.080) | (0.040) |
| Widowed | 0.030 | -0.052 | 0.045 | -0.048 |
|  | (0.094) | (0.057) | (0.095) | (0.057) |
| High School | -0.022 | 0.069 | -0.016 | 0.069 |
|  | (0.108) | (0.059) | (0.109) | (0.059) |
| Some College | 0.102 | 0.076 | 0.119 | 0.077 |
|  | (0.104) | (0.057) | (0.104) | (0.057) |
| Bachelor's Degree | 0.129 | 0.126* | 0.151 | 0.127* |
|  | (0.106) | (0.060) | (0.106) | (0.061) |
| Graduate School | 0.227* | 0.111 | 0.249* | 0.114 |
|  | (0.107) | (0.063) | (0.108) | (0.064) |
| Equivalized Household Income, $1000s | 0.000*** | 0.000 | 0.000*** | 0.000 |
|  | (0.000) | (0.000) | (0.000) | (0.000) |
| Health Insurance | -0.079 | 0.004 | -0.081 | 0.004 |
|  | (0.076) | (0.042) | (0.076) | (0.041) |
| Poor or Fair Health Status (binary) | -0.143* | 0.044 | -0.130* | 0.049 |
|  | (0.062) | (0.038) | (0.062) | (0.038) |
| Chronic Pain (binary) | -0.045 | 0.018 | -0.042 | 0.017 |
|  | (0.041) | (0.020) | (0.041) | (0.020) |
| C-reactive protein (z-score) | 0.010 | 0.000 | 0.014 | 0.002 |
|  | (0.019) | (0.010) | (0.018) | (0.010) |
| Fibrinogen (z-score) | -0.024 | 0.005 | -0.022 | 0.004 |
|  | (0.021) | (0.011) | (0.021) | (0.010) |
| Interleukin-6 (z-score) | -0.020* | 0.002 | -0.023** | 0.001 |
|  | (0.008) | (0.005) | (0.008) | (0.006) |
| E-Selectin (z-score) | -0.011 | -0.010 | -0.010 | -0.009 |
|  | (0.021) | (0.010) | (0.022) | (0.010) |
| Intercellular adhesion molecule-1 (z-score) | -0.001 | -0.002 | -0.001 | -0.002 |
|  | (0.014) | (0.008) | (0.014) | (0.008) |
| Neuroticism | -0.601*** | -0.114 | -0.594*** | -0.114 |
|  | (0.036) | (0.060) | (0.036) | (0.061) |
| Conscientiousness | 0.581*** | 0.115* | 0.585*** | 0.118* |
|  | (0.042) | (0.057) | (0.042) | (0.059) |
| Severe Psychological Distress (z-score) | -0.792*** | -0.016 | -0.782*** | -0.013 |
|  | (0.152) | (0.109) | (0.152) | (0.111) |
| Generalized Anxiety Disorder (binary) | -0.048 | -0.062 | -0.065 | -0.064 |
|  | (0.131) | (0.080) | (0.131) | (0.081) |
| Depressed Affect (M2) | -0.134 | 0.096* | -0.124 | 0.096* |
|  | (0.074) | (0.043) | (0.074) | (0.043) |
| 5+ Drinks (binary) | 0.007 | -0.001 | 0.007 | -0.001 |
|  | (0.008) | (0.004) | (0.008) | (0.004) |
| Substance Use (binary) | -0.018 | 0.036 | -0.011 | 0.035 |
|  | (0.059) | (0.030) | (0.059) | (0.030) |
| M2 | 0.092* | 0.036 | 0.096* | 0.036 |
|  | (0.039) | (0.021) | (0.039) | (0.021) |
| ACEs (z-score) | -0.067*** | - | -0.063*** | - |
|  | (0.017) |  | (0.017) |  |
| Daily Discrimination (z-score) | -0.076*** | - | -0.078*** | - |
|  | (0.021) |  | (0.021) |  |
| Constant | -0.821*** | -0.165 | -0.858*** | -0.174 |
|  | (0.209) | (0.134) | (0.210) | (0.138) |
|  |  |  |  |  |
| Montiel-Olea-Pfluegar Weak Instrument Test |  |  |  |  |
| Effective F | 16.83 |  | 16.04 |  |
| Critical Value: 5% Worst Case Bias | 5.14 |  | 4.97 |  |
| Overidentification Test |  |  |  |  |
| Hansen's J (p-value) | 0.399(0.53) |  | 0.601(0.44) |  |
| Endogeneity Test |  |  |  |  |
| Chi-squared (p-value) | 6.68(0.01) |  | 6.61(0.01) |  |
| Observations | 1,609 | 1,609 | 1,619 | 1,619 |
| Robust standard errors in parentheses |  |  |  |  |
| *** p<0.001, ** p<0.01, * p<0.05 |  |  |  |  |
| Two-stage Limited Information Maximum Likelihood (2SLIML) | |  |  |  |

# Supplementary Table S4 Effect of Flourishing (z-score) on Suicidal Ideation (binary) - Nonlinear Models

|  | (1) |  | (2) |  | (3) |  | (4) |  |
| --- | --- | --- | --- | --- | --- | --- | --- | --- |
|  | Flourishing  (z-score) |  | Suicidal Ideation (binary) |  | Flourishing  (z-score) |  | Suicidal Ideation (binary) |  |
| VARIABLES | First Stage |  | Second Stage |  | First Stage |  | Second Stage |  |
|  | IV Probit Coefficients | Standard Errors | IV Probit Coefficients | Standard Errors | IV Probit Coefficients | Standard Errors | IV Probit Coefficients | Standard Errors |
| Flourishing (Eudaimonic and Hedonic) (z-score) | - | - | -0.940*** | 0.209 | - | - | - | - |
| Flourishing (Eudaimonic only) (z-score) | - | - | - | - | - | - | -0.944*** | 0.212 |
| Ages 45-54 | 0.015 | 0.052 | 0.238* | 0.116 | 0.016 | 0.052 | 0.242* | 0.116 |
| Ages 55_64 | 0.055 | 0.056 | 0.080 | 0.112 | 0.052 | 0.056 | 0.085 | 0.112 |
| Ages 65 and over | 0.157** | 0.058 | 0.318** | 0.121 | 0.149** | 0.058 | 0.313** | 0.121 |
| Female | 0.132*** | 0.040 | 0.094 | 0.085 | 0.129*** | 0.040 | 0.090 | 0.085 |
| Asian/Pacific Islander | -0.078 | 0.221 | -0.271 | 0.421 | -0.074 | 0.221 | -0.270 | 0.418 |
| Black | 0.094 | 0.095 | 0.187 | 0.175 | 0.093 | 0.095 | 0.152 | 0.175 |
| Hispanic | 0.117 | 0.112 | 0.161 | 0.198 | 0.107 | 0.113 | 0.157 | 0.197 |
| Other Race | -0.052 | 0.079 | 0.077 | 0.171 | -0.056 | 0.080 | 0.073 | 0.171 |
| Married | 0.079 | 0.064 | -0.229 | 0.137 | 0.069 | 0.064 | -0.230 | 0.137 |
| Separated | 0.161 | 0.132 | -0.113 | 0.283 | 0.169 | 0.128 | -0.112 | 0.283 |
| Divorced | -0.020 | 0.080 | -0.331* | 0.147 | -0.021 | 0.080 | -0.324* | 0.146 |
| Widowed | 0.031 | 0.094 | -0.121 | 0.207 | 0.046 | 0.094 | -0.105 | 0.207 |
| High School | -0.023 | 0.107 | 0.271 | 0.250 | -0.017 | 0.108 | 0.266 | 0.249 |
| Some College | 0.101 | 0.103 | 0.276 | 0.242 | 0.118 | 0.103 | 0.280 | 0.240 |
| Bachelor's Degree | 0.128 | 0.104 | 0.486* | 0.249 | 0.150 | 0.105 | 0.489 | 0.247 |
| Graduate School | 0.226* | 0.106 | 0.413 | 0.257 | 0.247* | 0.107 | 0.421 | 0.256 |
| Equivalized Household Income, $1000s | 0.000*** | 0.000 | 0.000 | 0.000* | 0.000*** | 0.000 | 0.000* | 0.000 |
| Health Insurance | -0.080 | 0.075 | 0.009 | 0.159 | -0.082 | 0.075 | 0.007 | 0.157 |
| Poor or Fair Health Status (binary) | -0.144* | 0.061 | 0.096 | 0.133 | -0.131* | 0.062 | 0.112 | 0.133 |
| Chronic Pain (binary) | -0.045 | 0.040 | 0.088 | 0.086 | -0.043 | 0.040 | 0.084 | 0.085 |
| C-reactive protein (z-score) | 0.010 | 0.019 | -0.001 | 0.042 | 0.014 | 0.018 | 0.004 | 0.040 |
| Fibrinogen (z-score) | -0.023 | 0.021 | 0.016 | 0.045 | -0.021 | 0.021 | 0.011 | 0.044 |
| Interleukin-6 (z-score) | -0.020* | 0.008 | -0.003 | 0.016 | -0.023** | 0.008 | -0.006 | 0.019 |
| E-Selectin (z-score) | -0.011 | 0.021 | -0.040 | 0.040 | -0.011 | 0.021 | -0.038 | 0.040 |
| Intercellular adhesion molecule-1 (z-score) | -0.001 | 0.014 | -0.004 | 0.033 | -0.001 | 0.014 | -0.003 | 0.033 |
| Neuroticism | -0.600*** | 0.036 | -0.458** | 0.171 | -0.594*** | 0.036 | -0.456** | 0.171 |
| Conscientiousness | 0.581*** | 0.041 | 0.472** | 0.161 | 0.585*** | 0.042 | 0.481** | 0.164 |
| Severe Psychological Distress (z-score) | -0.796*** | 0.150 | -0.434 | 0.308 | -0.787*** | 0.150 | -0.422 | 0.310 |
| Generalized Anxiety Disorder (binary) | -0.049 | 0.129 | -0.213 | 0.235 | -0.066 | 0.130 | -0.223 | 0.233 |
| Depressed Affect (M2) | -0.134 | 0.073 | 0.226 | 0.154 | -0.123 | 0.073 | 0.227 | 0.154 |
| 5+ Drinks (binary) | 0.007 | 0.007 | -0.002 | 0.016 | 0.007 | 0.007 | -0.001 | 0.016 |
| Substance Use (binary) | -0.019** | 0.058 | 0.127 | 0.108 | -0.013 | 0.058 | 0.123 | 0.107 |
| M2 | 0.090* | 0.039 | 0.134 | 0.081 | 0.094* | 0.039 | 0.129 | 0.081 |
| ACEs (z-score) | -0.071*** | 0.015 | - | - | -0.068*** | 0.015 | - | - |
| Daily Discrimination (z-score) | -0.070*** | 0.021 | - | - | -0.070*** | 0.022 | - | - |
| Constant | -0.814*** | 0.206 | -2.174*** | 0.418 | -0.849*** | 0.206 | -2.190*** | 0.415 |
|  |  |  |  |  |  |  |  |  |
| ME: Continuous Flourishing (Eudaimonic and Hedonic) (z-score) | |  | -0.220** | 0.083 |  |  |  |  |
| ME: Continuous Flourishing (Eudaimonic only) (z-score) | |  |  |  |  |  | -0.222** | 0.086 |
| Observations | 1,609 |  | 1,609 |  | 1,609 |  | 1,609 |  |
| Robust Standard Errors |  |  |  |  |  |  |  |  |
| *** p<0.001, ** p<0.01, * p<0.05 |  |  |  |  |  |  |  |  |
| Instrumental Variable Probit Model (control function) | |  |  |  |  |  |  |  |
| ME: Marginal Effect |  |  |  |  |  |  |  |  |

# Supplementary Table S5 Effect of Flourishing (binary) on Suicidal Ideation (z-score) - Linear Models

|  | (1) | (2) | (3) | (4) |
| --- | --- | --- | --- | --- |
|  | Flourishing (binary) | Suicidal Ideation  (z-score) | Flourishing  (binary) | Suicidal Ideation  (z-score) |
| VARIABLES | First Stage | Second Stage | First Stage | Second Stage |
| Flourishing (Eudaimonic and Hedonic) (binary) | - | -0.959* | - | - |
|  |  | (0.449) |  |  |
| Flourishing (Eudaimonic only) (binary) | - | - | - | -0.928* |
|  |  |  |  | (0.428) |
| Ages 45-54 | -0.002 | 0.105 | -0.005 | 0.103 |
|  | (0.028) | (0.073) | (0.030) | (0.073) |
| Ages 55_64 | 0.046 | 0.061 | 0.038 | 0.055 |
|  | (0.031) | (0.070) | (0.033) | (0.069) |
| Ages 65 and over | 0.071* | 0.123 | 0.023 | 0.079 |
|  | (0.033) | (0.072) | (0.035) | (0.066) |
| Female | 0.041 | 0.021 | 0.048* | 0.026 |
|  | (0.023) | (0.052) | (0.024) | (0.053) |
| Asian/Pacific Islander | -0.147 | -0.336 | -0.170 | -0.360 |
|  | (0.115) | (0.174) | (0.128) | (0.184) |
| Black | 0.050 | 0.207 | 0.029 | 0.171 |
|  | (0.051) | (0.156) | (0.055) | (0.156) |
| Hispanic | 0.110 | 0.059 | 0.097 | 0.044 |
|  | (0.060) | (0.144) | (0.064) | (0.138) |
| Other Race | -0.062 | 0.037 | -0.060 | 0.038 |
|  | (0.042) | (0.135) | (0.045) | (0.132) |
| Married | 0.034 | -0.124 | 0.003 | -0.149 |
|  | (0.034) | (0.091) | (0.036) | (0.089) |
| Seperated | -0.031 | -0.396* | 0.021 | -0.336 |
|  | (0.066) | (0.182) | (0.079) | (0.203) |
| Divorced | -0.028 | -0.136 | -0.061 | -0.160 |
|  | (0.040) | (0.111) | (0.043) | (0.111) |
| Widowed | -0.024 | -0.033 | -0.011 | -0.018 |
|  | (0.059) | (0.172) | (0.060) | (0.170) |
| High School | -0.010 | 0.080 | 0.030 | 0.117 |
|  | (0.056) | (0.183) | (0.063) | (0.182) |
| Some College | 0.050 | 0.098 | 0.104 | 0.146 |
|  | (0.054) | (0.184) | (0.061) | (0.185) |
| Bachelor's Degree | 0.067 | 0.231 | 0.097 | 0.257 |
|  | (0.056) | (0.189) | (0.063) | (0.189) |
| Graduate School | 0.066 | 0.154 | 0.168** | 0.246 |
|  | (0.057) | (0.189) | (0.064) | (0.199) |
| Equivalized Household Income, $1000s | 0.000* | 0.000 | 0.000** | 0.000 |
|  | (0.000) | (0.000) | (0.000) | (0.000) |
| Health Insurance | -0.019 | 0.014 | -0.073 | -0.039 |
|  | (0.037) | (0.118) | (0.042) | (0.123) |
| Poor or Fair Health Status (binary) | -0.064* | 0.253* | -0.062 | 0.256* |
|  | (0.028) | (0.121) | (0.033) | (0.120) |
| Chronic Pain (binary) | -0.026 | 0.043 | -0.009 | 0.058 |
|  | (0.022) | (0.060) | (0.024) | (0.057) |
| C-reactive protein (z-score) | 0.012 | 0.008 | 0.004 | -0.000 |
|  | (0.011) | (0.023) | (0.010) | (0.022) |
| Fibrinogen (z-score) | -0.005 | 0.023 | -0.009 | 0.019 |
|  | (0.012) | (0.028) | (0.013) | (0.028) |
| Interleukin-6 (z-score) | -0.008 | -0.015 | -0.013** | -0.019 |
|  | (0.005) | (0.014) | (0.005) | (0.015) |
| E-Selectin (z-score) | -0.011 | -0.030 | -0.013 | -0.033 |
|  | (0.012) | (0.031) | (0.012) | (0.031) |
| Intercellular adhesion molecule-1 (z-score) | -0.002 | -0.006 | 0.007 | 0.003 |
|  | (0.011) | (0.025) | (0.010) | (0.027) |
| Neuroticism | -0.199*** | -0.136 | -0.216*** | -0.146 |
|  | (0.017) | (0.110) | (0.019) | (0.114) |
| Conscientiousness | 0.154*** | 0.099 | 0.178*** | 0.118 |
|  | (0.021) | (0.094) | (0.023) | (0.101) |
| Severe Psychological Distress (z-score) | 0.095* | 0.904** | 0.058 | 0.858* |
|  | (0.042) | (0.342) | (0.046) | (0.334) |
| Generalized Anxiety Disorder (binary) | 0.093** | 0.053 | 0.065 | 0.022 |
|  | (0.034) | (0.302) | (0.048) | (0.300) |
| Depressed Affect (M2) | 0.011 | 0.367** | -0.000 | 0.356** |
|  | (0.031) | (0.125) | (0.037) | (0.124) |
| 5+ Drinks (binary) | 0.003 | -0.008 | 0.001 | -0.010 |
|  | (0.004) | (0.009) | (0.004) | (0.009) |
| Substance Use (binary) | -0.048 | 0.089 | 0.012 | 0.143 |
|  | (0.028) | (0.090) | (0.032) | (0.085) |
| M2 | 0.011 | 0.046 | 0.037 | 0.066 |
|  | (0.022) | (0.053) | (0.023) | (0.055) |
| ACEs (z-score) | -0.044*** | - | -0.038*** | - |
|  | (0.010) |  | (0.010) |  |
| Daily Discrimination (z-score) | -0.022* | - | -0.040*** | - |
|  | (0.011) |  | (0.012) |  |
| Constant | 0.132 | -0.114 | 0.161 | -0.081 |
|  | (0.105) | (0.335) | (0.115) | (0.333) |
|  |  |  |  |  |
| Montiel-Olea-Pfluegar Weak Instrument Test |  |  |  |  |
| Effective F | 15.53 |  | 15.55 |  |
| Critical Value: 5% Worst Case Bias | 8.38 |  | 7.67 |  |
| Overidentification Test |  |  |  |  |
| Hansen's J (p-value) | 0.453(0.50) |  | 0.002(0.96) |  |
| Endogeneity Test |  |  |  |  |
| Chi-squared (p-value) | 5.31(0.02) |  | 5.44(0.02) |  |
| Observations | 1619 | 1619 | 1,619 | 1,619 |
| Robust standard errors in parentheses |  |  |  |  |
| *** p<0.001, ** p<0.01, * p<0.05 |  |  |  |  |
| Two-stage Limited Information Maximum Likelihood (2SLIML) | |  |  |  |

# Supplementary Table S6 Effect of Flourishing (z-score) on Suicidal Ideation (z-score) - Linear Models

|  | (1) | (2) | (3) | (4) |
| --- | --- | --- | --- | --- |
|  | Flourishing  (z-score) | Suicidal Ideation  (z-score) | Flourishing  (z-score) | Suicidal Ideation  (z-score) |
| VARIABLES | First Stage | Second Stage | First Stage | Second Stage |
| Flourishing (Eudaimonic and Hedonic) (z-score) | - | -0.518* | - | - |
|  |  | (0.229) |  |  |
| Flourishing (Eudaimonic only) (z-score) | - | - | - | -0.523* |
|  |  |  |  | (0.235) |
| Ages 45-54 | 0.014 | 0.113 | 0.015 | 0.116 |
|  | (0.052) | (0.070) | (0.052) | (0.070) |
| Ages 55_64 | 0.054 | 0.043 | 0.050 | 0.047 |
|  | (0.057) | (0.067) | (0.057) | (0.067) |
| Ages 65 and over | 0.156** | 0.137 | 0.147* | 0.136 |
|  | (0.059) | (0.073) | (0.059) | (0.073) |
| Female | 0.132** | 0.051 | 0.129** | 0.049 |
|  | (0.040) | (0.057) | (0.040) | (0.057) |
| Asian/Pacific Islander | -0.075 | -0.241 | -0.069 | -0.240 |
|  | (0.223) | (0.156) | (0.224) | (0.157) |
| Black | 0.100 | 0.217 | 0.101 | 0.194 |
|  | (0.096) | (0.158) | (0.096) | (0.155) |
| Hispanic | 0.116 | 0.011 | 0.107 | 0.010 |
|  | (0.113) | (0.129) | (0.114) | (0.129) |
| Other Race | -0.051 | 0.066 | -0.055 | 0.064 |
|  | (0.080) | (0.131) | (0.081) | (0.131) |
| Married | 0.077 | -0.115 | 0.067 | -0.116 |
|  | (0.065) | (0.092) | (0.065) | (0.091) |
| Seperated | 0.156 | -0.270 | 0.165 | -0.268 |
|  | (0.133) | (0.195) | (0.129) | (0.192) |
| Divorced | -0.023 | -0.118 | -0.025 | -0.115 |
|  | (0.081) | (0.105) | (0.080) | (0.105) |
| Widowed | 0.030 | 0.008 | 0.045 | 0.016 |
|  | (0.094) | (0.167) | (0.095) | (0.166) |
| High School | -0.022 | 0.083 | -0.016 | 0.081 |
|  | (0.108) | (0.183) | (0.109) | (0.183) |
| Some College | 0.102 | 0.109 | 0.119 | 0.111 |
|  | (0.104) | (0.182) | (0.104) | (0.183) |
| Bachelor's Degree | 0.129 | 0.243 | 0.151 | 0.246 |
|  | (0.106) | (0.187) | (0.106) | (0.188) |
| Graduate School | 0.227* | 0.215 | 0.249* | 0.220 |
|  | (0.107) | (0.191) | (0.108) | (0.194) |
| Equivalized Household Income, $1000s | 0.000*** | 0.000 | 0.000*** | 0.000 |
|  | (0.000) | (0.000) | (0.000) | (0.000) |
| Health Insurance | -0.079 | -0.014 | -0.081 | -0.013 |
|  | (0.076) | (0.118) | (0.076) | (0.117) |
| Poor or Fair Health Status (binary) | -0.143* | 0.234 | -0.130* | 0.245* |
|  | (0.062) | (0.119) | (0.062) | (0.119) |
| Chronic Pain (binary) | -0.045 | 0.046 | -0.042 | 0.044 |
|  | (0.041) | (0.057) | (0.041) | (0.056) |
| C-reactive protein (z-score) | 0.010 | 0.001 | 0.014 | 0.003 |
|  | (0.019) | (0.025) | (0.018) | (0.024) |
| Fibrinogen (z-score) | -0.024 | 0.020 | -0.022 | 0.016 |
|  | (0.021) | (0.028) | (0.021) | (0.027) |
| Interleukin-6 (z-score) | -0.020* | -0.017 | -0.023** | -0.019 |
|  | (0.008) | (0.013) | (0.008) | (0.014) |
| E-Selectin (z-score) | -0.011 | -0.027 | -0.010 | -0.027 |
|  | (0.021) | (0.029) | (0.022) | (0.029) |
| Intercellular adhesion molecule-1 (z-score) | -0.001 | -0.005 | -0.001 | -0.004 |
|  | (0.014) | (0.021) | (0.014) | (0.021) |
| Neuroticism | -0.601*** | -0.256 | -0.594*** | -0.256 |
|  | (0.036) | (0.157) | (0.036) | (0.159) |
| Conscientiousness | 0.581*** | 0.252 | 0.585*** | 0.258 |
|  | (0.042) | (0.152) | (0.042) | (0.156) |
| Severe Psychological Distress (z-score) | -0.792*** | 0.384 | -0.782*** | 0.393 |
|  | (0.152) | (0.353) | (0.152) | (0.354) |
| Generalized Anxiety Disorder (binary) | -0.048 | -0.068 | -0.065 | -0.073 |
|  | (0.131) | (0.292) | (0.131) | (0.292) |
| Depressed Affect (M2) | -0.134 | 0.293* | -0.124 | 0.291* |
|  | (0.074) | (0.133) | (0.074) | (0.132) |
| 5+ Drinks (binary) | 0.007 | -0.007 | 0.007 | -0.007 |
|  | (0.008) | (0.009) | (0.008) | (0.009) |
| Substance Use (binary) | -0.018 | 0.128 | -0.011 | 0.125 |
|  | (0.059) | (0.082) | (0.059) | (0.082) |
| M2 | 0.092* | 0.085 | 0.096* | 0.082 |
|  | (0.039) | (0.055) | (0.039) | (0.056) |
| ACEs (z-score) | -0.067*** | - | -0.063*** | - |
|  | (0.017) |  | (0.017) | - |
| Daily Discrimination (z-score) | -0.076*** | - | -0.078*** |  |
|  | (0.021) |  | (0.021) |  |
| Constant | -0.821*** | -0.655 | -0.858*** | -0.675 |
|  | (0.209) | (0.385) | (0.210) | (0.393) |
|  |  |  |  |  |
| Montiel-Olea-Pfluegar Weak Instrument Test |  |  |  |  |
| Effective F | 16.83 |  | 16.04 |  |
| Critical Value: 5% Worst Case Bias | 5.09 |  | 4.92 |  |
| Overidentification Test |  |  |  |  |
| Hansen's J (p-value) | 0.000(0.99) |  | 0.010(0.92) |  |
| Endogeneity Test |  |  |  |  |
| Chi-squared (p-value) | 4.05(0.04) |  | 4.07(0.04) |  |
| Observations | 1,609 | 1,609 | 1,619 | 1,619 |
| Robust standard errors in parentheses |  |  |  |  |
| *** p<0.001, ** p<0.01, * p<0.05 |  |  |  |  |
| Two-stage Limited Information Maximum Likelihood (2SLIML) | |  |  |  |
